# An Uncommon Focus of a Common Phenomenon: Superior Vena Cava Triggering Atrial Fibrillation

**DOI:** 10.19102/icrm.2023.14114

**Published:** 2023-11-15

**Authors:** Ammar Ahmed, Harini Lakshman, Steven Coutteau, Dipak Shah

**Affiliations:** 1 Department of Cardiovascular Disease, Ascension Providence Hospital, Southfield, MI, USA; 2 Abbott Laboratories, Abbott Park, IL, USA

**Keywords:** Ablation, atrial fibrillation, super vena cava

## Abstract

Ablation of atrial fibrillation most commonly involves the pulmonary veins; however, the superior vena cava (SVC) is an important potentially arrhythmogenic structure that should not be overlooked. This case report demonstrates an excellent example of triggering activity localized to the SVC and the subsequent conversion to sinus rhythm with ablation of the SVC.

## Introduction

Ablation for atrial fibrillation (AF) involves pulmonary vein (PV) isolation (PVI); however, it is important to consider non-PV sites for patients with refractory AF despite PVI. This case report highlights a clear instance of AF being triggered from the superior vena cava (SVC). Radiofrequency ablation (RFA) was successfully employed to isolate the SVC and restore sinus rhythm.

## Case description

A 61-year-old man with symptomatic paroxysmal AF and previous PVI was referred for repeat ablation. Mapping prior to the RFA revealed recovery of only the right inferior PV (RIPV). Given that it was his second ablation, the posterior wall was isolated in addition to reisolating the RIPV. Despite the subsequent ablation, he continued to have premature atrial contractions (PACs) and then atrial tachycardia and AF with burst pacing. Potential left atrial targets including the left atrial appendage and mitral annulus were excluded as triggers. The right atrium was then mapped.

**Figure 1:**
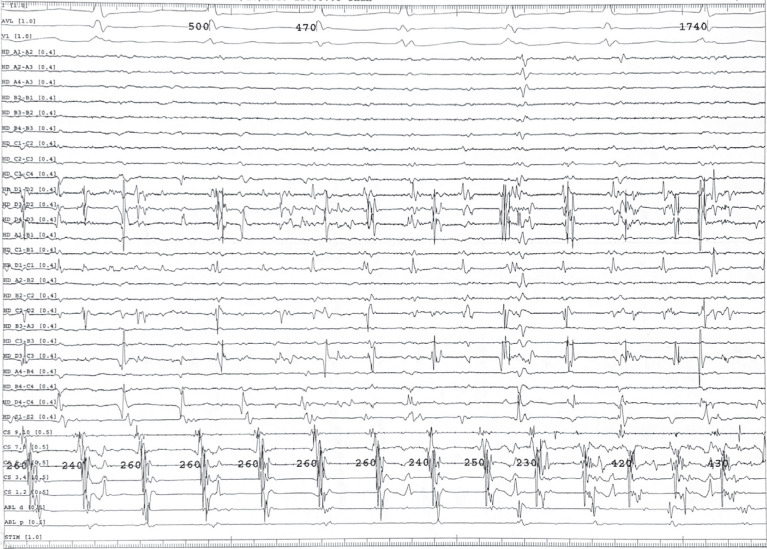
Electrogram illustrating AF. The Advisor™ HD Grid catheter was placed in the superior vena cava, which is likely the source of triggering activity considering the accelerated and chaotic signal relative to the coronary sinus signal.

**Figure 2:**
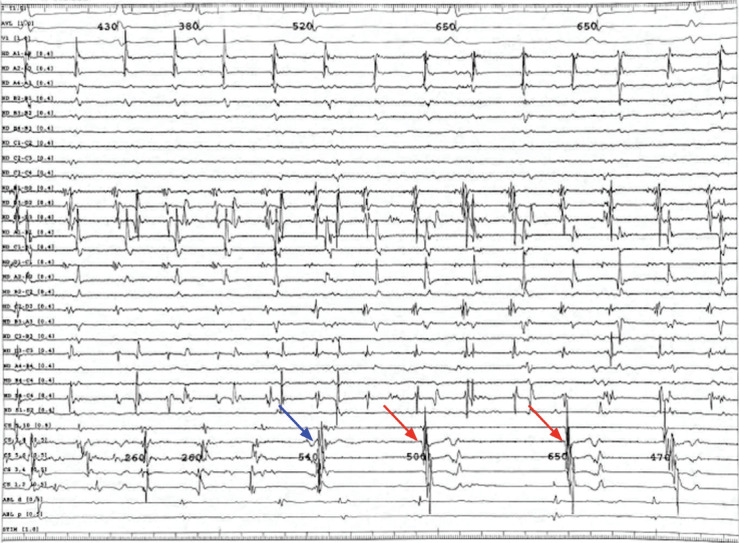
Atrial fibrillation at the beginning of superior vena cava isolation. During ablation, the patient converted to sinus rhythm (indicated by the coronary sinus catheter electrograms); however, the Advisor™ HD Grid catheter in the superior vena cava still found atrial fibrillation, indicating dissociation. The transition from the blue to red arrows indicates the conversion to sinus rhythm.

**Figure 3:**
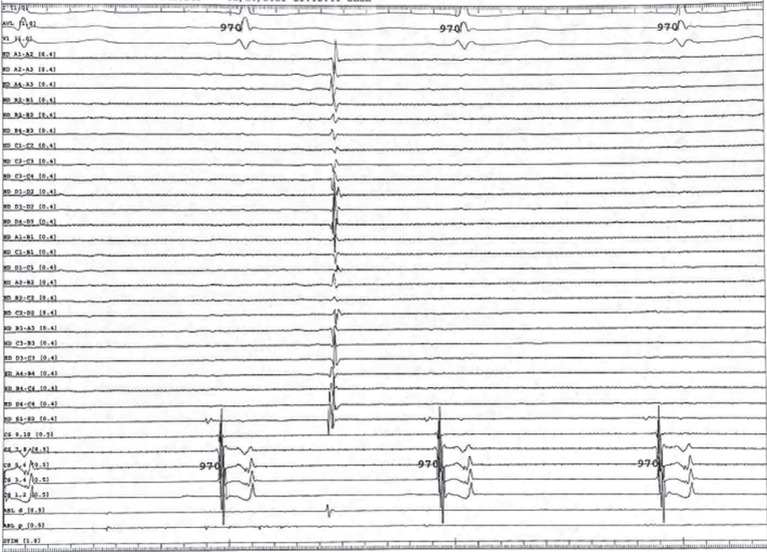
Coronary sinus signals showing sinus rhythm and HD Grid in the superior vena cava initially showing atrial fibrillation. During continued ablation, the superior vena cava terminated from atrial fibrillation and was electrically isolated.

With the Advisor™ HD Grid catheter (Abbott, Chicago, IL, USA) placed in the SVC, an accelerated and chaotic signal was seen relative to the coronary sinus (CS) **(Figure 1)**. As such, this was considered highly suspicious for triggering AF. Isolation of the SVC was performed after confirming no phrenic nerve capture at ablation targets. As ablation was occurring, the SVC initially remained in AF, but the patient then transitioned to normal sinus rhythm with PACs **(Figure 2)**. As the circumferential SVC isolation was completed, the SVC was no longer in AF, and the entrance and exit blocks were demonstrated with a dissociated potential **(Figure 3)**.

## Discussion

This case report clearly highlights electrograms (EGMs) triggering AF from the SVC and the progression of the EGMs and termination of AF with SVC isolation.

Although varying figures have been reported, an estimated 26%–28% of AF patients have foci outside the PVs.^1^ Of the non-PV foci, the SVC constitutes roughly 26%–40% and is the most common non-PV trigger site.^1,2^ Interestingly, patients with an arrhythmogenic SVC have less structural or systemic disease when compared to those with AF.^3^ Although the patient in our case did have coronary artery disease, it is valuable to have greater suspicion of an SVC trigger in patients who fit this profile. Additionally, coexistence of atrial flutter should increase suspicion, as this has also been associated with an SVC trigger.^3^ However, while it is important to consider the SVC as an arrhythmogenic source, trials have not found any benefit with empiric SVC ablation.^2^ Current practice remains to isolate the SVC when triggering foci are seen.^2^

Complication of SVC ablation must also be carefully considered. The most common complication involves injury to the right phrenic nerve, which courses in close proximity to the SVC.^4^ Injury is typically observed during ablation of the posterolateral SVC. Although injury results in paralysis of the right hemidiaphragm, most patients will recover within a year. Fortunately, injury can be avoided by pacing and demonstrating a lack of capture by the phrenic nerve prior to ablation. Additionally, SVC stenosis may result due to circumferential swelling with ablation also performed distal in the SVC.^4^

## Conclusion

We report a clear SVC trigger and the response to SVC isolation with corresponding EGMs. While PVs account for the majority of AF triggering activity, it is important to evaluate alternative sites in patients with refractory AF.
